# *De novo* transcriptome assembly of an Antarctic nematode for the study of thermal adaptation in marine parasites

**DOI:** 10.1038/s41597-023-02591-4

**Published:** 2023-10-19

**Authors:** Marialetizia Palomba, Pietro Libro, Jessica Di Martino, Xavier Roca-Geronès, Armando Macali, Tiziana Castrignanò, Daniele Canestrelli, Simonetta Mattiucci

**Affiliations:** 1 Department of Ecological and Biological Sciences, Tuscia University, Viale dell’Università s/n, 01100 Viterbo, Italy; 2 Department of Biology, Health and Environment, Section of Parasitology, Faculty of Pharmacy and Food Sciences, University of Barcelona, Joan XXIII Avenue, 27-31, 08028 Barcelona, Spain; 3 Department of Public Health and Infectious Diseases, Section of Parasitology, Sapienza University of Rome, P.le Aldo Moro, 5, 00185 Rome, Italy

**Keywords:** Evolutionary ecology, Computational biology and bioinformatics

## Abstract

Understanding the genomic underpinnings of thermal adaptation is a hot topic in eco-evolutionary studies of parasites. Marine heteroxenous parasites have complex life cycles encompassing a free-living larval stage, an ectothermic intermediate host and a homeothermic definitive host, thus representing compelling systems for the study of thermal adaptation. The Antarctic anisakid *Contracaecum osculatum* sp. D is a marine parasite able to survive and thrive both at very cold and warm temperatures within the environment and its hosts. Here, a *de novo* transcriptome of *C. osculatum* sp. D was generated for the first time, by performing RNA-Seq experiments on a set of individuals exposed to temperatures experienced by the nematode during its life cycle. The analysis generated 425,954,724 reads, which were assembled and then annotated. The high-quality assembly was validated, achieving over 88% mapping against the transcriptome. The transcriptome of this parasite will represent a valuable genomic resource for future studies aimed at disentangling the genomic architecture of thermal tolerance and metabolic pathways related to temperature stress.

## Background & Summary

Temperature is a critical factor in marine environments that plays a significant role in shaping the evolution of life^1,2^. In particular, fluctuations in temperature experienced in the marine realm pose significant challenges for the organisms. They have evolved numerous adaptations to maintain homeostasis across a wide thermal range, including modifications to membrane lipids, enzymes, and metabolic pathways, as well as changes in gene expression profiles^1,2^.

Recent advances in -omics sciences have allowed researchers to investigate the genomic foundations of thermal adaptation processes, which are highly relevant in evolutionary research. Through genome and transcriptome sequencing and assembly, valuable insights into the genetic mechanisms that enable diverse marine organisms to survive and thrive in environments with extreme conditions, have been gained^3–6^. However, while extensive research has been conducted on organisms that thrive in extreme temperatures, there is currently a notable gap in specific studies focusing on the molecular mechanisms which are at the base of thermal fluctuations.

Marine anisakid parasites, which are particularly subjected to fluctuations in temperatures due to their association with both ectothermic and/or homeothermic hosts^7^, provide a particularly interesting system for studying this phenomenon. These parasites employ a range of strategies, including heat-tolerant and freeze-avoiding mechanisms, to ensure their survival and adaptability^1^. The Antarctic anisakid nematode, *Contracaecum osculatum* sp. D inhabits the Earth’s coldest marine ecosystem, the Antarctic Sea^8^ and exhibits exceptional thermal adaptability. From the first larva to the adult stage, this parasite species experiences a range of temperatures. It is capable of surviving and thriving in both cold and warm temperatures during different stages of its heteroxenous life cycle. For instance, it undergoes a free-living larval stage, a third larval stage in ectothermic hosts (invertebrates and icefish), and the adult stage in homeothermic pinniped hosts (i.e. the Weddell Seal, *Leptonychotes weddellii*)^9^. As a consequence, *C. oscultum* sp. D may be exposed to subzero temperatures, likely exhibiting tolerance to thermal stress to maintain homeostasis, and finally possesses adaptive features to thrive to its suitable definitive host’s temperature.

Temperature can have significant effects on the development of the parasite in seawater, its relationship with the host, and the transmission of the parasite to its hosts. Temperatures experienced during different stages of its life cycle can directly or indirectly drive the population dynamics of the infection cycles of the parasite. Despite these challenges, the parasite species have been found to have a consistently high population size in its intermediate/paratenic and definitive hosts under a temporal scale level (from 1995 to 2014), indicating successful maintenance of parasites’ fitness through evolutionary adaptation’s features^8,9^.

The recent advance in -omic sciences of marine anisakid nematodes^10–17^ provides new opportunities for investigating the genetic basis of the evolutionary traits that underlie the adaptation of these heteroxenous parasites to their hosts.

Therefore, the aim of the present study was to provide a transcriptomic resource for investigating the genetic underpinnings in terms of disclosing putative genes and metabolic pathways involved in the thermal adaptation speculated by the Antarctic parasite *C. osculatum* sp. D through its life cycle.

The resource generated by the present comprehensive analysis will provide a deeper understanding of the evolutionary forces that have shaped the genomic architecture of thermal-adapted marine organisms, particularly in these anisakid parasites, and provide valuable insights into their survival strategies in the challenging marine environment. Moreover, these advances in -omic sciences hold great promise for uncovering the molecular basis of thermal adaptation in marine organisms and for promoting conservation efforts aimed at protecting marine Antarctic ecosystems, considering the ongoing oceans ‘environmental change.

## Methods

### Sample collection and RNA preparation

*Contracaecum osculatum* (sensu lato) third-stage larvae (L3) were extracted from the body cavity of the ice fish, *Chionodraco hamatus* caught in the Ross Sea, Antarctica. The parasitological examination was conducted on-site, specifically at the Zucchelli Station in the Ross Sea, during the expedition financed by the Italian PNRA-MUR 2019 (National Antarctic Research Program-Ministry of University and Research). The careful removal was done using scissors and tweezers. Then, L3 were examined for their integrity under a dissecting microscope, and their vitality was assessed based on their spontaneous movements. Alive and not disrupted larvae were washed, following the procedure, as previously reported^17^. Larvae were then cultured under different thermal profiles, which are believed to mimic the temperature conditions experienced by the Antarctic anisakid during its life cycle (i.e., −2 °C, 1 °C, 37 °C) (Table 1). At specific time intervals, N = 54 L3 were promptly preserved in RNAlater solution until further analyses. RNA and DNA were extracted from whole L3 using TRIzol reagent (Invitrogen, Carlsbad, CA, USA), according to the manufacturer’s instructions with some modifications as previously reported^18^. DNA was used to identify L3 at the species level following procedures previously reported^8^. RNA was treated with DNase (DNase I, Invitrogen) according to the manufacturer’s instructions. Subsequently, RNA from each group of three individuals, belonging to the same experimental condition, was pooled together. The quality and concentration of RNA were evaluated using a spectrophotometer (NanoDrop® 8000, Thermo Fisher Scientific, US) and a Bioanalyzer (Agilent 2100, Agilent Technologies, Santa Clara, USA).

**Table 1:** Summary of the 18 libraries deposited in the Sequence Read Archive (SRA) of NCBI (SRP422483)^43^.

Run ID	Temperature	Raw sequence	Filtered sequence	% trimmed read
SRR23456143	−2 °C	22,634,131	20,736,790	91,62
SRR23456144	−2 °C	21,025,371	18,647,738	88,69
SRR23456145	−2 °C	24,914,886	22,018,652	88,38
SRR23456146	−2 °C	25,226,687	23,011,308	91,22
SRR23456147	−2 °C	25,192,198	23,000,985	91,30
SRR23456157	−2 °C	23,345,551	21,297,927	91,23
SRR23456158	−2 °C	23,026,402	20,808,470	90,37
SRR23456148	1 °C	22,162,833	23,000,985	89,26
SRR23456149	1 °C	22,937,773	20,536,345	89,53
SRR23456150	1 °C	20,049,583	18,077,263	90,16
SRR23456159	1 °C	22,387,210	20,237,154	90,40
SRR23456160	1 °C	20,747,424	18,508,542	89,21
SRR23456156	37 °C	33,435,950	29,964,089	89,62
SRR23456155	37 °C	20,858,439	19,085,468	91,50
SRR23456154	37 °C	24,951,744	22,987,951	92,13
SRR23456153	37 °C	25,963,075	23,158,637	89,20
SRR23456152	37 °C	24,076,721	21,753,688	90,35
SRR23456151	37 °C	23,018,746	20,657,143	89,74

For each sample (from three whole L3) is reported the run ID, exposure temperature, number of raw, filtered and trimmed reads.

### Library preparation and sequencing

mRNA sequencing libraries were prepared using the Illumina Truseq stranded mRNA library prep kit, according to the manufacturer’s instructions. Briefly, mRNA was purified and fragmented from total RNA using poly-T oligo-attached magnetic beads. The resulting cleaved RNA fragments, primed with random hexamers, were reverse transcribed into first-strand cDNA using reverse transcriptase, random primers, and dUTP instead of dTTP. The incorporation of dUTP halts the second strand during amplification since the polymerase cannot extend beyond this nucleotide. The generated cDNA fragments were further processed by adding a single ‘A’ base and ligating the adapter. The resulting products were then purified and enriched using PCR to generate a final strand-specific cDNA library. The quality of the amplified libraries was assessed using capillary electrophoresis (Bioanalyzer, Agilent).

After performing qPCR using the SYBR Green PCR Master Mix (Applied Biosystems), the libraries with index tags in equimolar amounts were pooled. The cluster generation step took place in the flow cell using the cBot automated cluster generation system (Illumina). Subsequently, the flow cell was loaded onto the NovaSeq 6000 sequencing system (Illumina) for sequencing, employing a read length of 2 × 100 base pairs (bp). The sequencing data are available at the NCBI Sequence Read Archive (Table 1).

### Pre-assembly processing stage

**Fig. 1:**
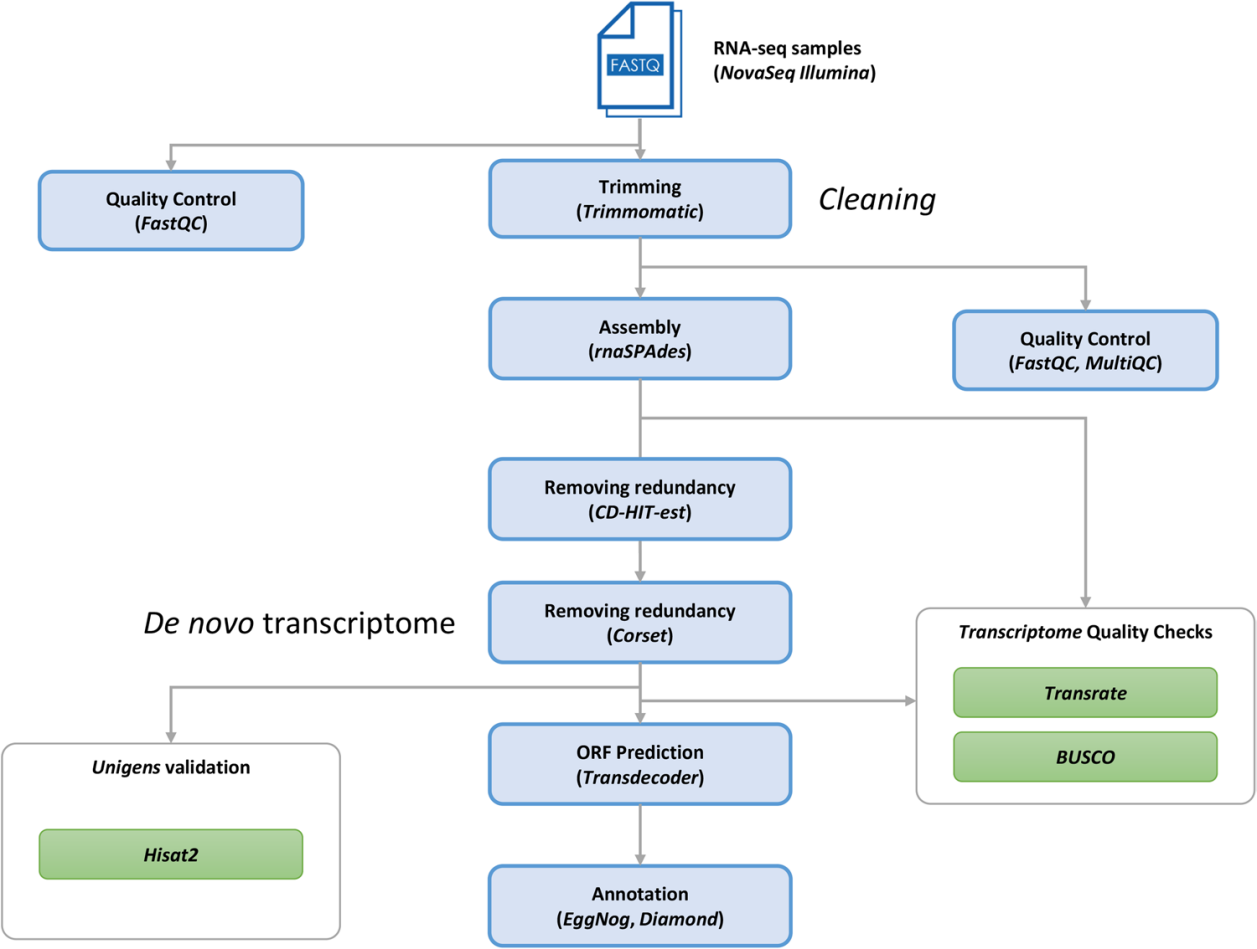
Workflow of the bioinformatic pipeline for the *de novo* transcriptome assembly of *Contracaecum osculatum* sp. D, starting from raw data and leading to annotated scripts.

The obtained RNA-seq data on *C. osculatum* sp. D L3 were processed for mass transcriptome sequencing. The workflow of the bioinformatics pipelines, adapted from two previous studies^19,20^, is illustrated in Figure 1. The bioinformatic analyses were conducted using the High-Performance Computing resources supplied by ELIXIR-IT HPC@CINECA^21–25^. A total of 425,954,724 pairs of reads were generated. All of them underwent a cleaning and analytic step. Read quality control was performed by running FastQC (v. 0.11.5) (http://www.bioinformatics.bbsrc.ac.uk/projects/fastqc), before and after the trimming phase. Detailed quality results are freely available in the figshare archive (Table 2). Quality evaluation metrics of the trimmed data were aggregated for all samples into a single report for concise visualisation using the software tool MultiQC (v. 1.9)^26^. Raw reads underwent a quality trimming phase using Trimmomatic (v. 0.39) to remove low-quality bases and adapter sequences. The Trimmomatic parameters were as follows: SLIDINGWINDOW:4:15, MINLEN:36, and HEADCROP:13)^27^. Unpaired reads were also discarded for the rest of the assembly pipeline. After the cleaning and removal of low-quality reads, a total of 387,489,135 reads were retained for de novo transcriptome assembly, corresponding to 92% of the raw reads (Table 1).

**Table 2:** Overview of produced data files and their access on figshare^45^.

Label	Name of data	Data repository (URL)
Image file 1	Per sequence quality scores (MultiQC)	10.6084/m9.figshare.23012903
Image file 2	Mean quality scores (MultiQC)	10.6084/m9.figshare.23012825
Data file 1	rnaSPAdes RNA-seq *de novo* transcriptome assembly	10.6084/m9.figshare.22298557
Data file 2	CD-HIT-est output (Filtered)	10.6084/m9.figshare.22298545
Data file 3	Corset output (Filtered)	10.6084/m9.figshare.22298524
Data file 4	Predicted ORFs (Cds)	10.6084/m9.figshare.22298815
Data file 5	Predicted ORFs (Proteins)	10.6084/m9.figshare.23522820
Data file 6	BLASTX vs NR	10.6084/m9.figshare.22298836
Data file 7	BLASTX vs SwissProt	10.6084/m9.figshare.22298863
Data file 8	BLASTX vs TrEMBL	10.6084/m9.figshare.22298866
Data file 9	BLASTP vs NR	10.6084/m9.figshare.22298872
Data file 10	BLASTP vs SwissProt	10.6084/m9.figshare.22298875
Data file 11	BLASTP vs TrEMBL	10.6084/m9.figshare.22298878
Data file 12	Eggnog output	10.6084/m9.figshare.23012549
Data file 13	*A. pegreffii* transcriptome (Corset)	10.6084/m9.figshare.23465780
Data file 14	*A. pegreffii* predicted ORFs	10.6084/m9.figshare.23465084
Data file 15	*A. simplex* (s.s.) transcriptome	10.6084/m9.figshare.23739111
Data file 16	*A. simplex* (s.s.) predicted ORFs	10.6084/m9.figshare.23465330
Data file 17	Orthogroups (OrthoFinder)	10.6084/m9.figshare.23465525

### *De novo* transcriptome assembly and quality assessment

Given the unavailability of a reference genome for *C. osculatum* sp. D, a *de novo* transcriptome assembly procedure was carried out. To ensure the construction of an optimized transcriptome and avoid chimera transcripts, we used rnaSPAdes^28^, a tool for *de novo* transcriptome assembly implemented in the SPAdes package (v. 3.14.1). RnaSPAdes automatically detected two k-mer sizes, approximately one-third and half of the maximum read length (the detected k-mer sizes were 45 and 67 nucleotides, respectively). A total of 237,314 assembled contigs were generated from rnaSPAdes runs with an N50 of 1783 bp (Table 3). Two filtering steps were performed to accurately remove assembly redundancies. The first step was performed by launching CD-HIT-est (v. 4.8.1) on the rnaSPAdes output; the result was uploaded on figshare (Table 2). The final assembly was produced by Corset (v. 1.06)^29^, a tool we employed based on its successful application in a previous study^30^. The Corset output showed an N50 of 1871 bp (Table 3).

**Table 3:** Statistics on rnaSPAdes and Corset outputs evaluated with the Transrate assembly validator.

Validation scores	rnaSPAdes output	Corset output (unigenes)
**Basic parameters**		
Total transcripts	237,314	46,690
N50	1783	1871
GC content (%)	0,39	0,37
**Transrate v. 1.0.3**		
Transrate Assembly Score	0,0294	0,0759
Transrate Optimal Score	0,0403	0,0812
Transrate Optimal Cutoff	0,012	0,0434
Good contigs	217,098	42,621
p good contigs	0,91	0,91

Thanks to the two-step process of removing redundancies, which effectively reduces assembly chimeras and improves the accuracy of subsequent analyses, the final assembly contained about 20% of the original transcripts.

**Fig. 2:**
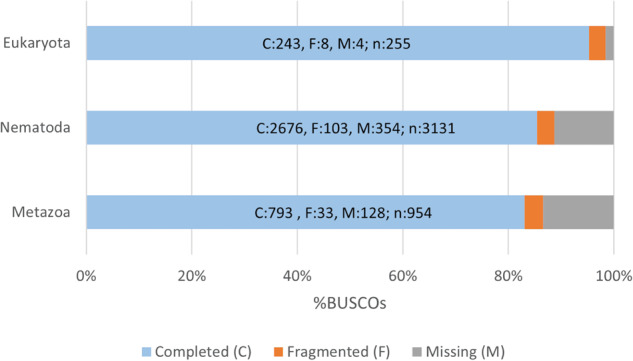
BUSCO assessment results.

The validation process encompassed two distinct phases applied to the assembly outcomes. The initial phase aimed at evaluating the preliminary assembly, followed by another phase aimed at appraising the quality of the final, non-redundant assembly. Two distinct tools were employed: TransRate (v. 1.0.3)^31^ and BUSCO (Benchmarking Universal Single-Copy Orthologs) (v. 5.4.4)^32^. These tools generated an array of metrics, serving as a valuable compass for identifying potential errors within the assembly process and offering evidence about the quality of the de novo assembled transcriptome. The TransRate results (Table 3) also include ‘good contigs’ values, which represent the number of contigs in the assembly classified as high quality by the validator, and the ‘p good contigs’ value, which indicates the percentage of high quality contigs compared to the total number of contigs in the assembly. BUSCO provides a quantitative measure of transcriptome quality and completeness, founded on evolutionarily derived predictions of gene content from databases housing nearly universal and ultra conserved protein orthologs. The analysis of gene content was carried out by conducting BUSCO assessments on three orthologous gene databases: Nematoda, Metazoa, and Eukaryota. The completeness of the transcriptome by BUSCO is reported in Table 4. Figure 2 illustrates completed, fragmented and missing genes mapped from the three databases.

**Table 4:** The BUSCO (v. 5) validation, through the gVolante web server, was applied to three databases: Nematoda, Metazoa and Eukariota.

Busco Category	Nematoda	Metazoa	Eukaryota
Complete BUSCOs (C)	2676 (85,47%)	793 (83,12%)	243 (95,30%)
Complete and single-copy BUSCOs (S)	1703 (54,40%)	603 (63,10%)	192 (75,30%)
Complete and duplicated BUSCOs (D)	970 (31,00%)	190 (20,00%)	51 (20,00%)
Fragmented BUSCOs (F)	103 (3,30%)	33,39 (3,50%)	8 (3,10%)
Missing BUSCOs (M)	354 (11,30%)	128 (13,40%)	4 (1,60%)
Total BUSCO groups searched	3131	954	255

### Generation of the full-length transcriptomes

**Fig. 3:**
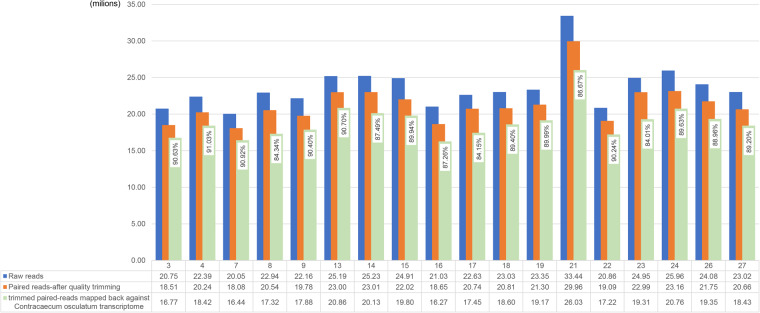
For each sample, the representation of the total paired-reads is shown in blue, the total paired-reads after removal of the adapters and quality trimming is shown in orange, and the trimmed paired-reads mapped against the *de novo* assembled *Contracaecum osculatum* sp. D transcriptome is shown in green.

After the validation and evaluation phase, the resulting data from the assembly process serves as the input for the CD-HIT-est program^33^. This hierarchical clustering tool is employed to circumvent redundancy among transcripts and to address fragmented assemblies in the *de novo* assembly process, yielding unique genes. CD-HIT-est was executed with default settings, resulting in a 95% similarity threshold. To refine the final transcriptome dataset, an additional hierarchical clustering phase was performed using Corset. This groups related transcripts based on their expression patterns, effectively identifying and merging isoforms, and transcriptional variants. This phase significantly improves the accuracy and completeness of the transcriptome assembly. Subsequently, the output of Corset was validated by BUSCO, and quality assessment was conducted using HISAT2 (v. 2.1)^34^ by mapping the trimmed reads to the reference transcriptome (unigenes). HISAT2 results showed a percentage of at least 88% (Fig. 3), providing the relative fraction of RNA-seq reads used to assemble the transcriptome.

The results of all validation phases are shown in Table 2 and discussed in the “Technical Validation” section.

The Corset output was run with TransDecoder (v. 5.7.0)^35,36^, a current standard tool for identifying long open read frames (ORFs) within assembled transcripts, using default parameters. TransDecoder performs ORF prediction on both transcript strands, irrespective of the sequenced library. Additionally, it evaluates ORF completeness and discerns potential 5′ end incompleteness by detecting any length of amino acid (AA) codons downstream of a start codon (M) without a stop codon. The “Longest ORF” criterion was employed, leading to identify the earliest 5' AUG codon as the start site for translation.

### Transcriptome annotation

We employed different kinds of annotations for the *de novo* assembly. We introduced DIAMOND^37^, an open-source algorithm based on double indexing that is 20,000 times faster than BLASTX on short reads and has a similar degree of sensitivity. Like BLASTX, DIAMOND attempts to determine exhaustively all significant alignments for a given query. Most sequence comparison programs, including BLASTX, follow the seed-and-extend paradigm. In this two-phase approach, users search first for matches of seeds (short stretches of the query sequence) in the reference database, and this is followed by an ‘extend’ phase that aims to compute a full alignment. The following parameter settings were applied: DIAMOND-fast DIAMOND BLASTX-t 48 -k 250 -min-score 40; DIAMOND-sensitive: DIAMOND BLASTX -t 48 -k 250 -sensitive -min-score 40.

Contigs were aligned with DIAMOND against the NCBI non-redundant (NR) protein database, which includes the non-redundant RefSeq proteins. In addition, the SwissProt and TrEMBL databases were also used to retrieve the best matching annotations for contigs. An annotation matrix was then generated by selecting the best hit for each database.

After conducting the BLASTX analysis against Nr, TrEMBL and SwissProt, we obtained annotations for 29,694 (80,3%), 29,904 (80,9%) and 20,660 (55,9%) contigs, respectively. Similarly, using BLASTP *versus* Nr, TrEMBL and SwissProt, we annotated 24,366 (65,9%), 24,600 (66,5%) and 17,239 (46,6%) contigs, respectively.

All the information on the resulting datasets is summarised in Table 5. The overview of data files and data sets produced in this study are summarised in Table 2.

**Table 5:** Summary of homology annotation hits on the different databases queried in this study.

Database	Number of BLASTX results	Number of BLASTP results
Nr	29,694 (80,30%)	24,366 (65,90%)
TrEMBL	29,904 (80,90%)	24,600 (66,50%)
SwissProt	20,660 (55,90%)	17,239 (46,60%)

**Fig. 4:**
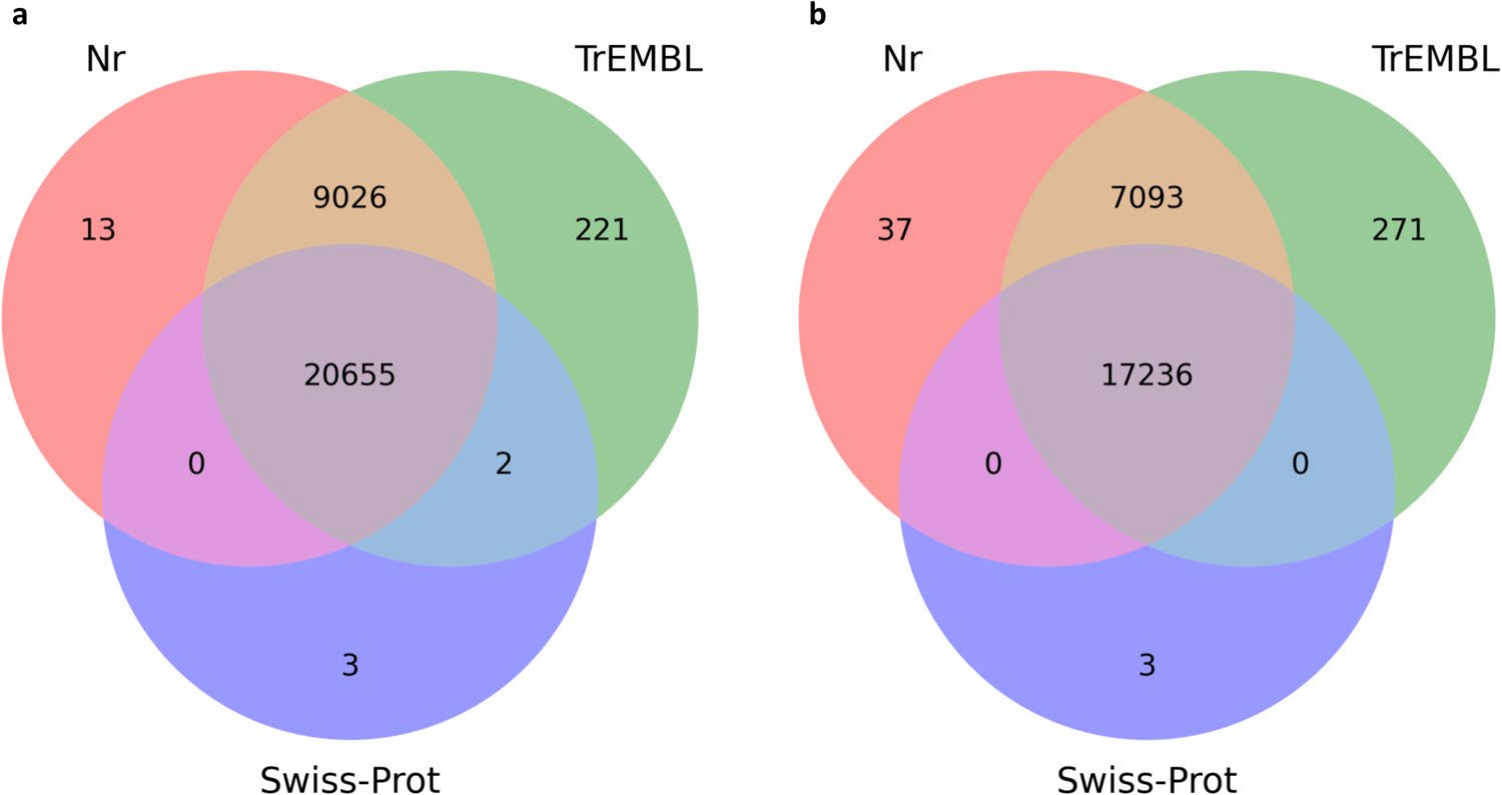
Venn diagrams for the number of contigs annotated with DIAMOND (BLASTX (**a**) and BLASTP (**b**) functions) against the three databases: Nr, SwissProt, TrEMBL.

**Fig. 5:**
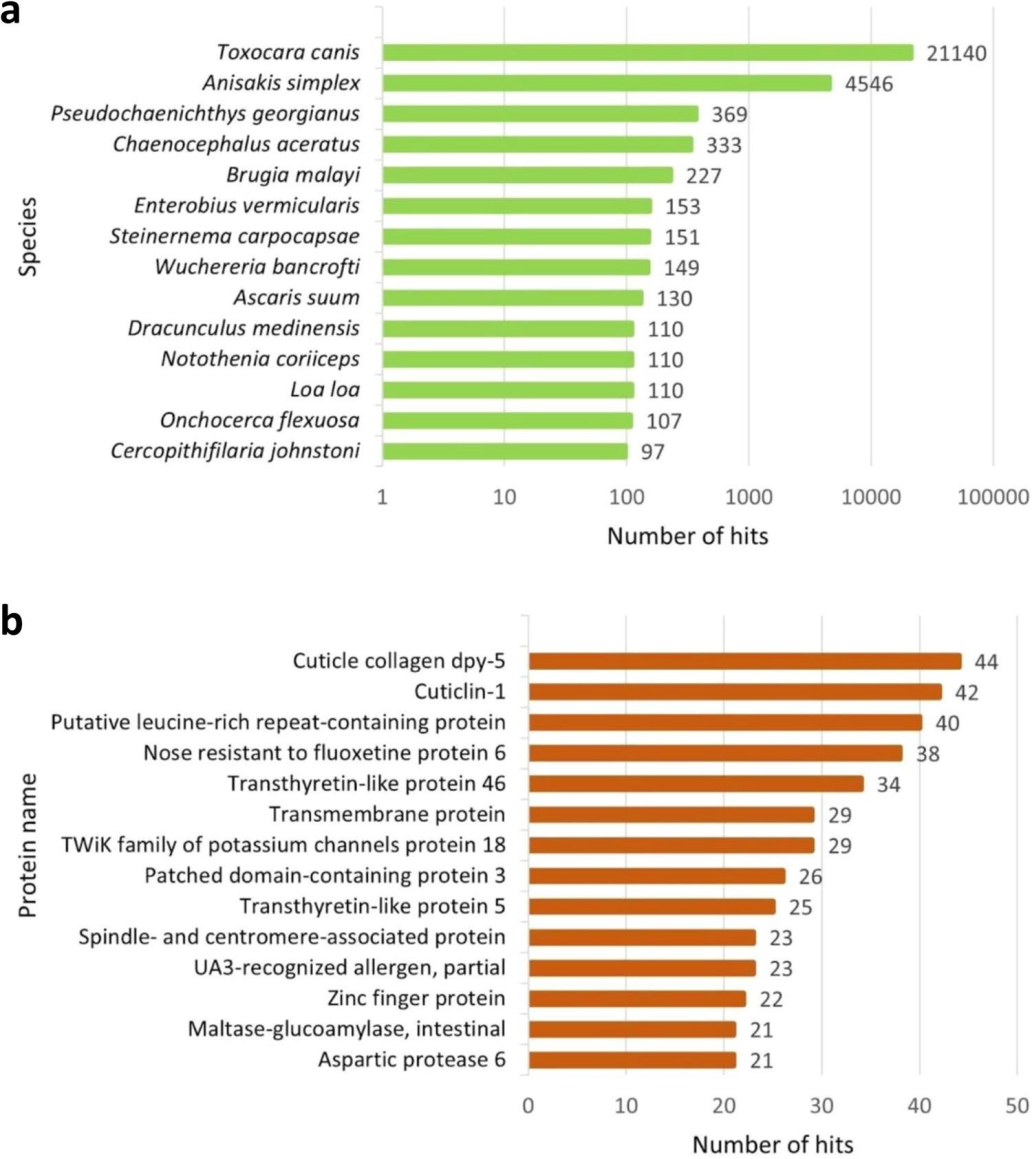
Most represented species and gene product hits. Top 10 best species (**a**) and protein (**b**) hits present in the reference database (Nr, BLASTX).

**Fig. 6:**
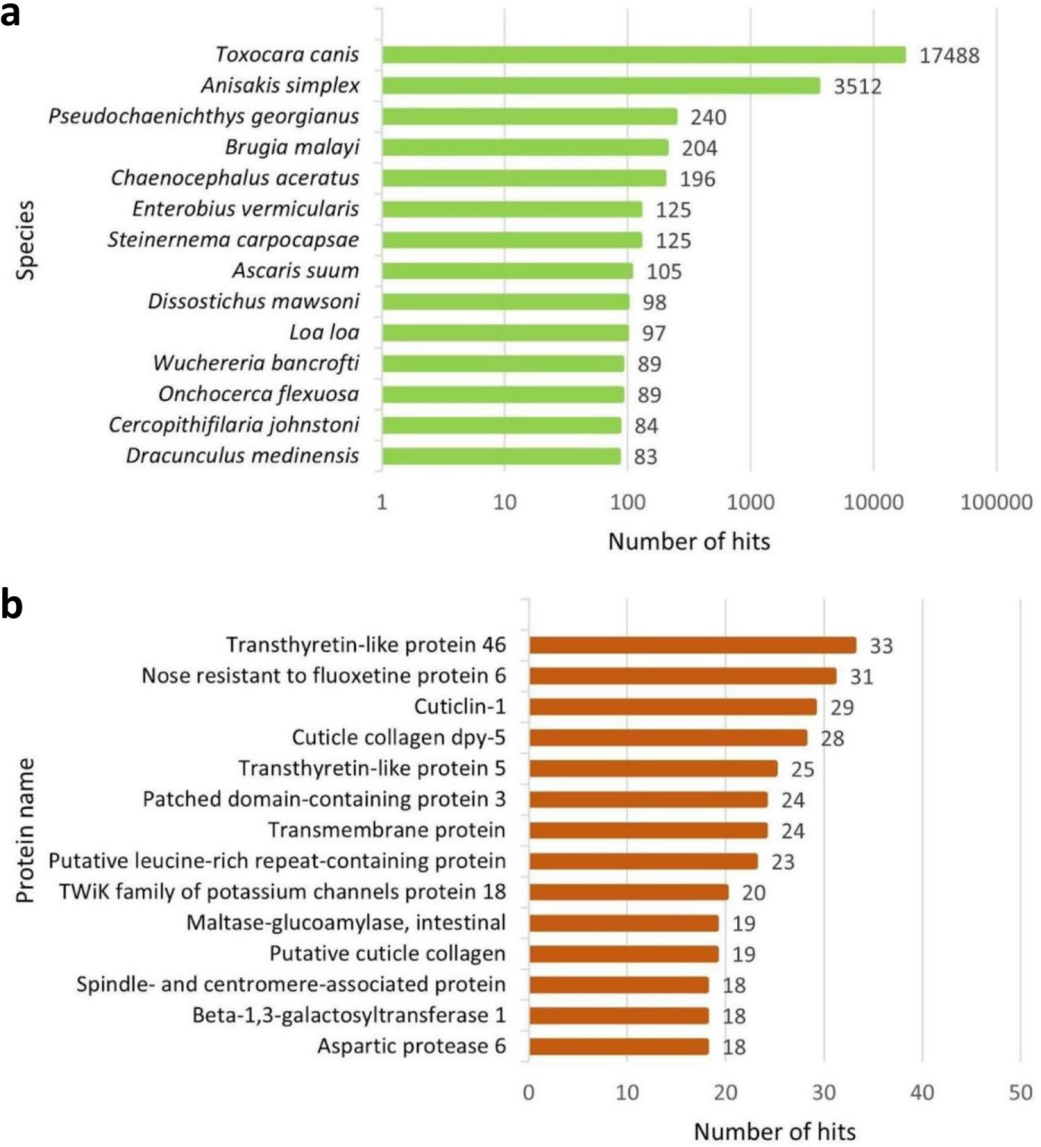
Most represented species and gene product hits. Top 10 best species (**a**) and protein (**b**) hits present in the reference database (Nr, BLASTP).

The output obtained from the BLASTX annotation consisted of a total of 20,655 sequences mapped simultaneously to the three interrogated databases (i.e., Nr, SwissProt and TrEMBL). The output from the BLASTP annotation consisted of a total of 17,236 sequences mapped simultaneously to the three databases. Venn diagrams are presented in Fig. 4, showing the redundancy of the annotations in the different databases for both DIAMOND BLASTX (Fig. 4a) and DIAMOND BLASTP (Fig. 4b). Furthermore, the ten most represented species and the ten hits of the gene product obtained respectively with BLASTX and BLASTP by mapping the transcripts against the reference database Nr are shown in Figures 5, 6. The BLASTX approach translates nucleotide sequences in all six frames (three forward and three reverse) into protein sequences before conducting the search against protein databases, making it more exhaustive than the BLASTP approach, which directly aligns protein sequences against protein databases.

The total number of unigenes obtained from the transcriptome assembly was also mapped onto another database of functional annotations: EggNOG (Evolutionary genealogy of genes: Non-supervised Orthologous Groups)^38^. The EggNOG database incorporates various taxonomic levels of orthologous groups (OG) of proteins with functional annotations, using an algorithm that builds on previous orthologous group (COG) methodologies. This database offers detailed functional information for genes within each orthologous group and includes a wide range of sequenced genomes from different species, providing a robust evolutionary context for our data analysis. Of the 36,985 total predicted ORFs, 16,968 (or 45,9%) were annotated in the EggNOG database. For details, see Table 2.

### Comparison with closest species through the orthologs

**Fig. 7:**
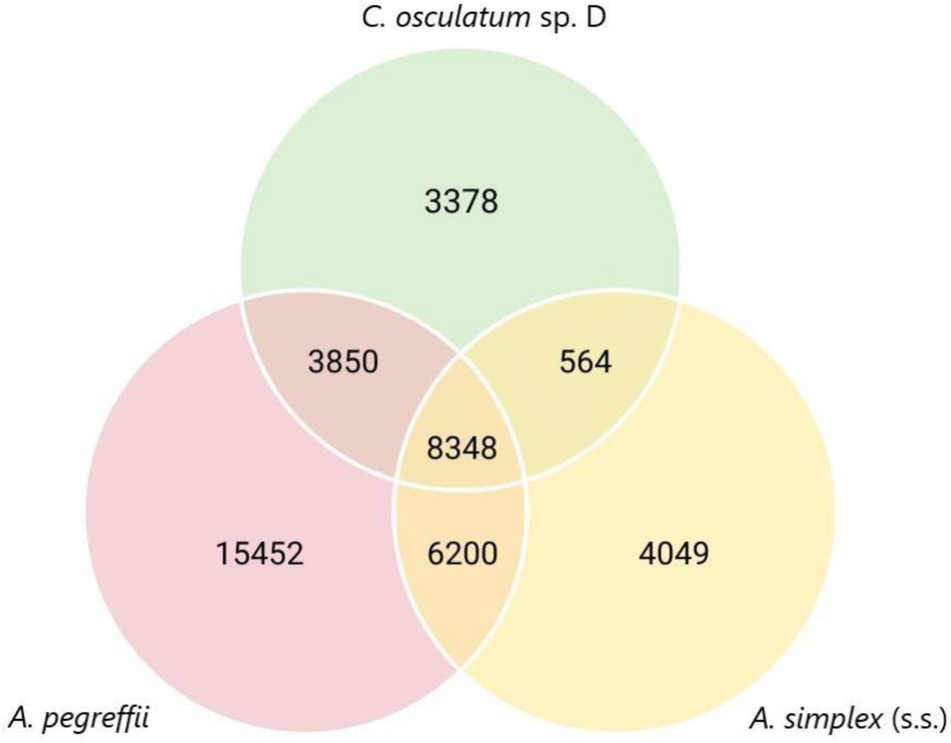
Venn diagram representing the number of species-specific and overlapping protein orthogroups between the three transcriptomes. The number of orthogroups were identified with OrthoFinder.

We compared the predicted ORFs from the *de novo* transcriptome of *C. osculatum* sp. D with both the predicted ORFs of the transcriptome of *Anisakis pegreffii*^16^ and the transcripts of *A. simplex* (sensu stricto)^39^. The reference transcriptome of *A. simplex* (s.s.) was produced with GffRead^40^, an open-source program to manipulate GFF and GTF format files. The identification and orthological grouping of all the proteins of the three species were performed using OrthoFinder (v. 2.5.5)^41^. This approach also served to assess the completeness of the assembly based on sequence similarity. OrthoFinder allows orthogroup detection, defined as a set of genes descended from a single gene of the last common ancestor within species groups^42^. The orthogroup detection demonstrated considerable overlap in transcript sequences in all three groups: *A. pegreffii*, *A. simplex* (s.s.) and *C. osculatum* sp. D. More than 20% (8348) of the transcripts identified as putative orthologs were shared between all three species (Fig. 7). We found that 15452 transcripts (36,9%) in *A. pegreffii*, 4049 transcripts (9,7%) in *A. simplex* (s.s.), and 3378 transcripts (8,1%) in *C. osculatum* sp. D were classified as species-specific. Thus, the marked level of sequence overlap observed between transcriptomes further validates the completeness and quality of the assembly presented in this study.

## Data Records

All raw data generated in this project have been deposited in National Center for Biotechnology Information Sequence Read Archive (NCBI SRA, PRJNA934921)^43^. The *de novo* transcriptome assembly resource was deposited on figshare (Table 2) and NCBI (GKNQ00000000)^42^ after Foreign Contamination Screen (FCS). All files produced in the transcriptome assembly and annotation were deposited in the figshare archive (Table 2).

## Technical Validation

The data quality was assessed using FastQC, pre and post trimming analysis. In the FastQC results, the average quality scores at every base position exceeded the threshold of 35 (Image file 1, Table 2). Validation of the transcriptome assembly was performed using two validation tools: BUSCO and TransRate. The results of validation processes are presented in Table 3. BUSCO analysis was performed on three databases: Nematoda, Metazoa and Eukariota. The details of BUSCO are listed in Table 4. Complete (C), Missing (M) and Fragmented (F) genes are plotted in Figure 2. An additional validation assessment was performed by mapping the clipped reads against the* de novo* assembled transcriptome of *C. osculatum* sp. D using the HISAT2 tool (Fig. 3). To further assess the quality of transcriptome assembly, the number of full-length assembled transcripts was evaluated^44^. The outcomes reveal a total of 5188 proteins, which exhibit coverage exceeding 90% of their respective protein lengths. The final transcriptome (unigenes) after contamination screening comprised a total of 43,673 transcripts and an N50 of 1867 bp. The BUSCO evaluation attested a completeness over 80% for each database interrogated.

### Quality control of annotation

Functional annotation of the transcriptome was accomplished by DIAMOND and EggNOG. The application of DIAMOND for annotation purposes led to the identification of 20,655 predicted ORFs (for BLASTX analyses) and 17,236 predicted ORFs (for BLASTP analyses) shared between the three databases used: Nr, SwissProt and TrEMBL. Finally, from the EggNOG analysis, we obtained COG (Cluster of Orthologous Groups) annotations and KEGG (Kyoto Encyclopedia of Genes and Genomes) annotations for 16,968 ORFs, representing 45,9% of the total.

## Data Availability

The article includes a comprehensive list of software programs employed for various tasks, such as *de novo* transcriptome assembly, pre- and post-assembly procedures, and transcriptome annotation, all of which are specified alongside their respective versions within the Methods section. If specific parameter details are not provided, the programs were used with their default settings.
